# The impacts of intrauterine Bisphenol A exposure on pregnancy and expression of miRNAs related to heart development and diseases in animal model

**DOI:** 10.1038/s41598-020-62420-1

**Published:** 2020-04-03

**Authors:** Zatilfarihiah Rasdi, Roziana Kamaludin, Sharaniza Ab. Rahim, Syed Baharom Syed Ahmad Fuad, Mohd Hafiz Dzarfan Othman, Rosfaiizah Siran, Noor Shafina Mohd Nor, Narimah Abdul Hamid Hasani, Siti Hamimah Sheikh Abdul Kadir

**Affiliations:** 1 Institute of Medical Molecular Biotechnology, Faculty of Medicine, Universiti Teknologi MARA, Cawangan Selangor, 47000 Sungai Buloh, Selangor Malaysia; 2 Centre of Preclinical Sciences Studies, Faculty of Dentistry, Universiti Teknologi MARA, Cawangan Selangor, 47000 Sungai Buloh, Selangor Malaysia; 3 Advanced Membrane Technology Research Centre (AMTEC), Universiti Teknologi Malaysia, 81310 Skudai, Johor Malaysia; 4 Faculty of Medicine, Universiti Teknologi MARA, Cawangan Selangor, 47000 Sungai Buloh, Selangor Malaysia; 5 Institute for Pathology, Laboratory and Forensic Medicine (I-PPerForM), Universiti Teknologi MARA, Cawangan Selangor, 47000 Sungai Buloh, Selangor Malaysia

**Keywords:** Biochemistry, Developmental biology, Environmental sciences, Molecular medicine

## Abstract

This study aimed to examine the impact of BPA exposure on pregnancy and foetuses on cardiac tissues and the expression of cardiac microRNAs (miRNAs) related to heart development and diseases. Pregnancy is known to be the “critical windows” in determining the offspring physical and cells development in their life after birth. The increment of the risk of cardiovascular disease (CVD) in a later stage of life has been reported by few studies demonstrated from prenatal exposure of BPA. BPA has been shown to alter miRNAs expression profiles for organ development, regeneration and metabolic functions. These alterations have been associated with the risk of CVDs. However, the associations between pregnancy outcomes and miRNAs expression in cardiac of mother- and foetuses-exposed to BPA are still not entirely explored. In BPA-exposed pregnant rat groups, a significant weight gained was observed in comparison to control (p < 0.05). Interestingly, significant changes in systolic and diastolic blood pressure between the first and third trimester of BPA-exposed pregnant rats were also observed (p < 0.05). In BPA-exposed pregnant rats, miR-499-5p was significantly altered in the heart (p < 0.01). Meanwhile, altered miR-17-5p, -208-3p, and -210-3p expressions were observed in all heart of the foetuses from BPA-exposed pregnant rats (p < 0.05). In H&E staining, BPA-exposed foetal hearts showed a sign of fibrosis while BPA-exposed pregnant rats showed muscle remnant. Masson trichrome staining further confirmed the presence of fibrosis observed in BPA-exposed foetal heart and reduced expression of cardiac troponin I (cTnI) was also observed in BPA-exposed foetal heart. In summary, altered cardiac miRNAs with histological changes were observed in both mother- and foetus-exposed BPA These findings put forward the importance of future work to further understand how prenatal BPA exposure affect foetuses in their later stage of life.

## Introduction

Altered foetal development “programming” may predispose certain individuals to the risk of chronic disease development later in their life. This was suggested by Barker and co-worker who presented the first finding on the increased risk of cardiovascular disease (CVD) in children of malnourished mothers^1^. Barker then further extended his theory and linked the CVD development and insulin resistance to the environment of the placenta. His findings have attracted another study to report on the insufficiency of uteroplacental of malnutrition mothers increases the risk of the offspring to type 2 diabetes^2^. Another study demonstrated that miR-208, a cardiac-specific miRNA, plays a role in the development of cardiac hypertrophy and fibrosis in response to stress^3^. Moreover, miR-133a was also found to be upregulated in the plasma of CVD patients^4^. Remarkably, these findings have led the scientist to associate miRNAs expression with the risk of heart disease development later in life.

Another study has demonstrated the importance of miR-1 and miR-133a in foetal heart development as both miRNAs are expressed in heart disease subjects and are required during organogenesis^5^. The study also reported the role of miR-208 in regulating myosin heavy chain (MHC) switching. The disruption of this gene may result in the activation of the fast-skeletal muscle gene program within the heart. Interestingly, miR-21 expression is typically altered following cardiac stresses and is detectable in almost every tissue throughout the body^6^. Apart from that, miR-499 was found to be elevated in the experimental animal models of infarction and human patients. This shows the potential usage of miR-499 as a biomarker for infarction^7^.

Bisphenol A (BPA) is ubiquitously used in the industrial sectors, hence humans are exposed to the compound repetitively^8–10^. Although there are BPA-free products, however, the compound is still widely found in many consumer products. The contaminants are able to leach from those consumer products into the environment such as into water^11^, food^12^ and air^13^. Studies showed a significant level of urinary BPA were detected in BPA-related industrial workers and children^12,14,15^. In addition, BPA has also been detected in human serum with a concentration up to 4.4 ng/ml^16^ and 11.2 ng/ml. However, concentration as high as 11.2 ng/ml has been reported to be harmful for embryo development^17^. Furthermore, epidemiological studies have reported the association of BPA exposure with the development of metabolic diseases, especially CVD^18–22^. They demonstrated the association of higher urinary BPA levels with an increased risk of coronary artery disease (CAD), hypertension, and myocardial infarction.

Although BPA is rapidly bio-transformed in the adults, previous studies have shown that during pregnancy, BPA could reach the foetus via placenta^23^, thus, potentially could affect the development of the foetal organs including brain^24^, heart^18^, liver^25^, lungs^26^ and ovary^27^. In relation to Barker’s hypothesis, recent evidence showed that exposure of BPA in the intrauterine environment in mice has altered glucose homeostasis and served as the risk factor for CVD and diabetes development in the offspring^28^. BPA exposure also alters miRNA expression in the female reproductive system^29^, liver^30^, heart^31^, placenta^32^ and others. miR-146a was found to be overexpressed in the placental cells leading to slower proliferation, thus, increasing the potential of DNA damage. Moreover, miR-192 has been reported to be downregulated in hepatic steatosis that triggers non-alcoholic liver disease^30^. Another interesting finding was conducted by Bhaskaran *et al*. who found the important role of miR-127 in the foetal lung development^33^.

On the basis of the above discoveries, we hypothesised that BPA exposure during intrauterine environment could alter the expression of miRNAs, thus increasing the possibility of CVD development in the offspring. This study was conducted to explore the effects of intrauterine BPA exposure to foetuses cardiac tissues and miRNAs that is important for cardiac development.

## Main Text

### Materials and methods

#### Chemicals

Tween-80 and BPA were purchased from Sigma Aldrich. In this study, Tween-80 was used as vehicle control for treatment (with 0.4% of Tween-80 in total solution). BPA was dissolved in Tween-80 (with the same volume) and prepared to 20 ppm concentrations. For liver histology standard protocol, paraformaldehyde (PFA) and phosphate buffer solution (PBS) were used and were purchased from Sigma Aldrich and Thermo Scientific, respectively.

#### Animals experimentation

Adult female rats aged six to eight weeks (Sprague Dawley) (weighing between 200–250 g, n = 5) were housed individually and allowed free access for food and drinks. Females were mated with males rat in ratio 1:1 in different cages. The vagina smears were collected the next morning to confirm positive mating^34^. The pregnant rats were grouped into three categories namely drinking water containing vehicles (0.4% Tween-80^35^), water containing 5 ppm BPA and 20 ppm BPA (0.05 mg/ml and 0.2 mg/ml, respectively). Glass water bottles were used to avoid potential contamination from sources other than administration. The water intake was noted with an estimation of consumption approximately 25 mL or more per day. The treatment began on pregnancy day 2 (PD2) until pregnancy day 21 (PD21). The treatment began at PD2 to observe any significant effect on organogenesis^36^. This study was conducted in accordance with relevant guidelines and regulations and is approved by Universiti Teknologi MARA (UiTM) Committee of Animal Research and Ethics (Approval Number UiTM CARE: 222/7/2017 (8/12/2017)).

Throughout the treatment period, the weight of the pregnant rats was taken four times (on PD2, PD7, PD14 and PD21) whereas blood pressure (BP) was recorded twice during early (PD2) and end (PD18) of the pregnancy. BP readings were taken twice to minimise stress factor in the treated rats as it may contribute to number of factors such as miscarriage. Apart from that, lighting and temperature of the animal room were also monitored and controlled. Food intake was measured four times throughout the treatment period in which each pregnant rat was supplied with approximately 230 g of normal diet pellet.

Non-invasive BP measurement on the pregnant rats were conducted using volume pressure recording (VPR) sensor technology according to the methodology described by Wang *et al*.^37^. The rats were placed in a strainer with the nose protruded through the front of the nose cone to allow comfortable breathing while the tail was fully extended and exited through the rear hatch opening of the holder. The blood flow and blood volume in the tail were measured by VPR system and generated BP readings simultaneously.

Caesarean section was performed on PD21 according to the previous protocol^38^. Each litter of rats were weighed, and the size was measured. The number of foetuses for each rat was recorded (n = 8–12 pups/rat). Both pregnant and foetuses heart were isolated, placed in RNAlater buffer (QIAGEN, Dusseldorf, Germany) and stored in 4 °C. The samples were then stored at −80 °C on the following day. The livers were collected from both the mothers and the foetuses for histology and examination (H&E).

#### Liver histology and examination

Lobes of the mothers and foetal liver were fixed in 4% PFA in 0.1 M PBS, dehydrated, and embedded in paraffin. The livers were sectioned with 5 µm sections and transferred to microscope slides (Fisher Scientific, Whitby, ON). Sections were stained using the standard H&E protocol for histological analyses as described in a previous study^39^.

#### Heart histology and examination

Mothers heart were cut longitudinally while the total heart of the foetuses was used and fixed in formalin, dehydrated and embedded in paraffin. The hearts were sectioned with 5 µm sections and transferred to microscope slides (Fisher Scientific, Whitby, ON). Sections were stained using H&E protocol for histological analyses as described in a previous study^39^.

Sections were also stained for Masson Trichrome (MT) and immunofluorescence staining. For Masson Trichrome, the 5 µm sections of fetal hearts were deparaffined and stained according to the Masson Trichrome kit (Bio-Optica, Milano) prior to view.

Immunofluorescence staining was done by blocking the sections of fetal heart for 30 min with 1% horse serum in PBS, then incubated at room temperature for 3 hrs with primary antibody of troponin (1:400). Sections were washed thrice with PBS for 5 min and secondary antibody incubation was carried out using 1:500 fluorescein isothiocyanate (FITC). Subsequently they were washed thrice with PBS in 5 min. Tissues were counterstain with DAPI for 1 min, washed as previous. Tissues then were mounted with prolong gold antifade reagent and cover slipped prior to view under fluorescence microscope (Olympus, New York) and confocal microscope (Leica, UK)^40^.

#### miRNA extraction

In brief, the isolated samples were removed from RNAlater buffer (QIAGEN, Dusseldorf, Germany) and homogenised using a mortar and pestle^41^. miRNA was extracted from the heart samples using miRNeasy mini kit (QIAGEN, Dusseldorf, Germany). Approximately, three to four foetal hearts from each group or 40 mg of homogenised tissue was used for extraction. Extracted RNA was quantified using a Nanodrop spectrophotometer and aliquoted into single-use aliquots and stored at −80 °C. Total RNA was extracted from ∼40 mg of heart tissue using the AllPrep miRNA Universal Kit following the manufacturer’s instructions.

#### miRNA polymerase chain reaction array (miRNA-PCR)

Total RNA of 250 ng from tissue was used for cDNA synthesis using miScript RT II cDNA Synthesis Kit (QIAGEN, Dusseldorf, Germany) as per manufacturer’s protocols. miRNAs abundance was measured by miRNA PCR array using Applied Biosystems Fast Advanced Master Mix (QIAGEN, Dusseldorf, Germany). PCR was performed and the target genes were normalised to three chosen reference genes. SNORD68, SNORD96A and RNU6–6P were employed as the reference genes to analyse miRNAs expression in the rat cardiac tissues^42–44^. The ratio between the target gene and the geometric mean of the reference genes were calculated to achieve mean normalised expression (MNE) of the target genes in each sample. For analyses, Ct values > 35 cycles were excluded from the analyses if duplicates had a standard deviation greater than 1 cycle. Final probe concentrations were maintained as suggested by the manufacturer in a 20 μl reaction. Sample concentrations were 1:10 dilution of the stock pre-amplification generated product as per the manufacturer’s protocol. Covariation (CV) of the reference genes was determined from Biorad CFX Manager 3.1 that generate the stability values. CV value is an indicator of variation in a group, the less CV value showed low variation among the groups. From the data, the CV values of both reference genes of rat and foetus cardiac tissues were below 0.5, which indicates the stability of these genes is accurate and reasonable. miRNA PCR data was analysed using 2-ΔΔCT method.

The list of targeted miRNAs and their ID number were as tabulated in Table 1.

**Table 1:** Targeted miRNA and their manufactured ID number accession.

miRNA	ID number	NCBI Accession ID	Role of miRNA
rno-miR-15b-3p	MS00026831	MIMAT0017093	Newborn heart development
rno-miR-21-5p	MS00013216	MIMAT0000790	Disease biomarker and cardiac development
rno-miR-17-5p	MS00013118	MIMAT0000786	Disease biomarker
rno-miR-133a-3p	MS00033208	MIMAT0000839	Organogenesis, heart failure
rno-miR-208a-3p	MS00033292	MIMAT0000880	Cardiac specific miRNA
rno-miR-499-5p	MS00001169	MIMAT0003381	Myocardial infarction
rno-miR−210-3p	MS00000644	MIMAT0000881	Hypoxia and heart disease
rno-miR-30a-5p	MS00013363	MIMAT0000808	Cardiac muscle injury
SNORD68	MS00033712	NR_002450	Reference gene
SNORD96A	MS00033733	NR_002592	Reference gene
RNU6-6P	MS00033740	NR_002752	Reference gene

#### Statistical analysis

Non-parametric analysis was carried out using SPSS version 20 (SPSS, Inc.). Kruskal Wallis test was used where appropriate to compare control against BPA-exposed values with alpha set at p ≤ 0.05. Data are shown as mean ± SEM in miRNA expression while other data are shown as mean ± SD.

### Results

#### BPA has no effect on the mothers and foetuses liver

**Figure 1:**
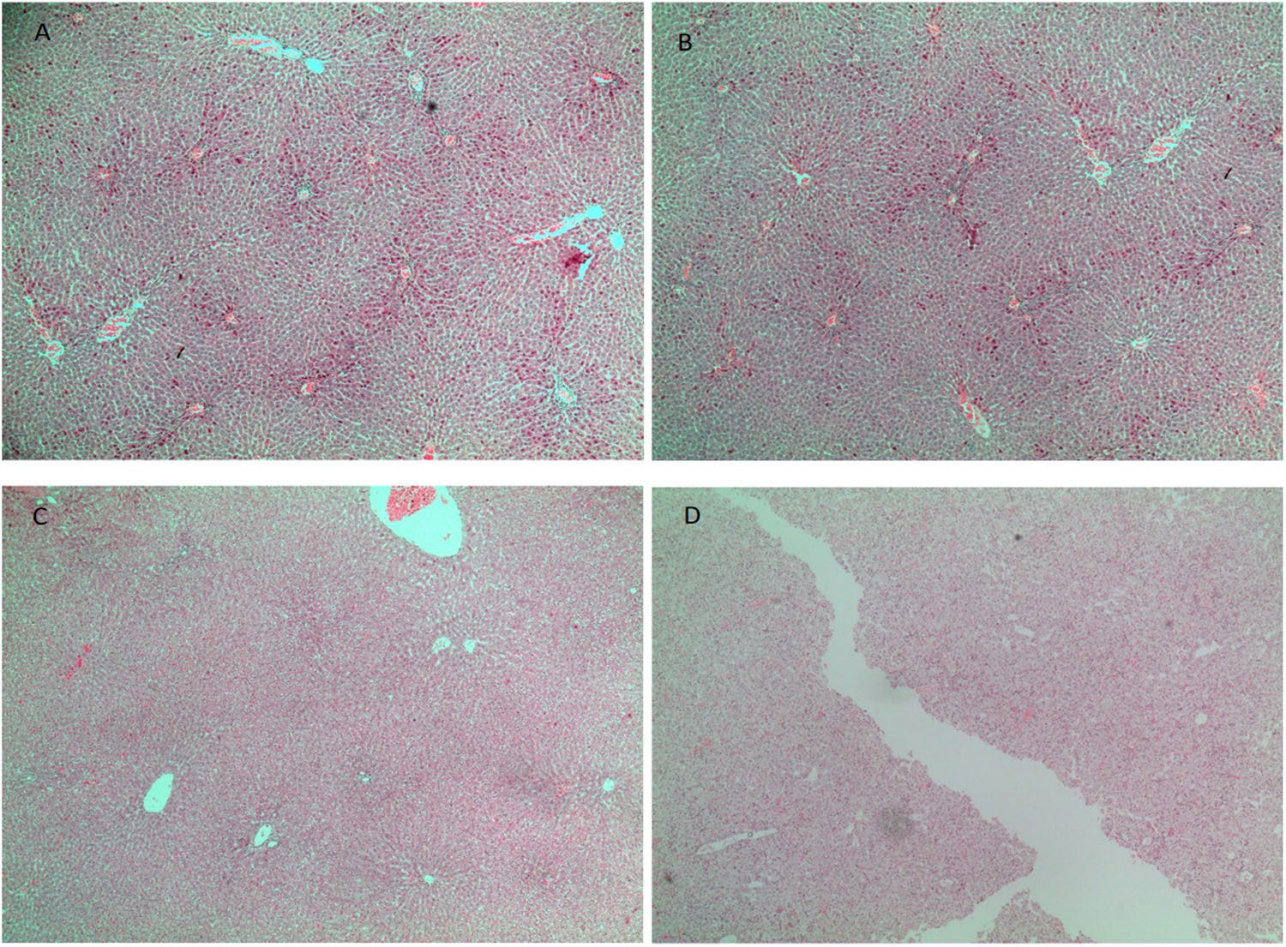
BPA shows no effect on mothers and foetuses liver histology. Representative of liver section of control (**A**) and BPA-exposed (**B**) pregnant rat, Foetal liver section at PD21 from pregnant rats of control (**C**) and BPA-exposed (**D**) stained with hematoxylin and eosin dye (10X magnification).

Standard histology of the liver was performed to determine the effect of prenatal BPA exposure to structural liver maturation of foetus from PD2 to PD18. Upon observation, we found that there were no visible differences in the structure between control and BPA-exposed in both pregnant and foetal liver (Fig. 1).

The H&E-stained liver sections of the control and BPA-exposed rats revealed normal liver histology, displaying a normal cell size, with a prominent cell nucleus, uniform cytoplasm, and radially aligned sinusoid and central vein. The similar structure pattern in the control and BPA-exposed foetus liver indicated that prenatal BPA exposure from PD2 to PD18 has no toxicity effect on the liver.

#### Exposure of BPA affects the histology of BPA-exposed pregnant rats and foetal heart

**Figure 2:**
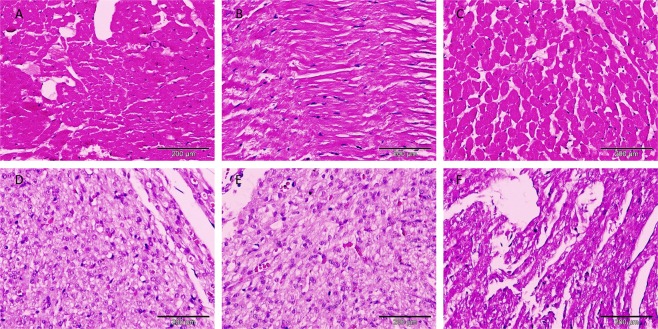
BPA exposure effects mothers and foetal hearts histology. Representative of heart section of control (**A**) and 5ppm and 20ppm BPA-exposed (**B**,**C**) pregnant rats, Foetal heart section at PD21 from pregnant rats of control (**D**) and 5 ppm and 20 ppm BPA-exposed (**E**,**F**) stained with haematoxylin and eosin dye (40X magnification).

Histology of the mother’s and foetus heart was performed to compare the morphology of the muscle cells in control and BPA-exposed subjects (Fig. 2). The H&E heart sections showed a sign of fibrosis in the BPA-exposed foetus heart. Meanwhile, in the mothers, the heart of the BPA-exposed pregnant rats showed muscle remnants.

#### BPA exposure during pregnancy affects the mothers’ weight gained and blood pressure

**Figure 3:**
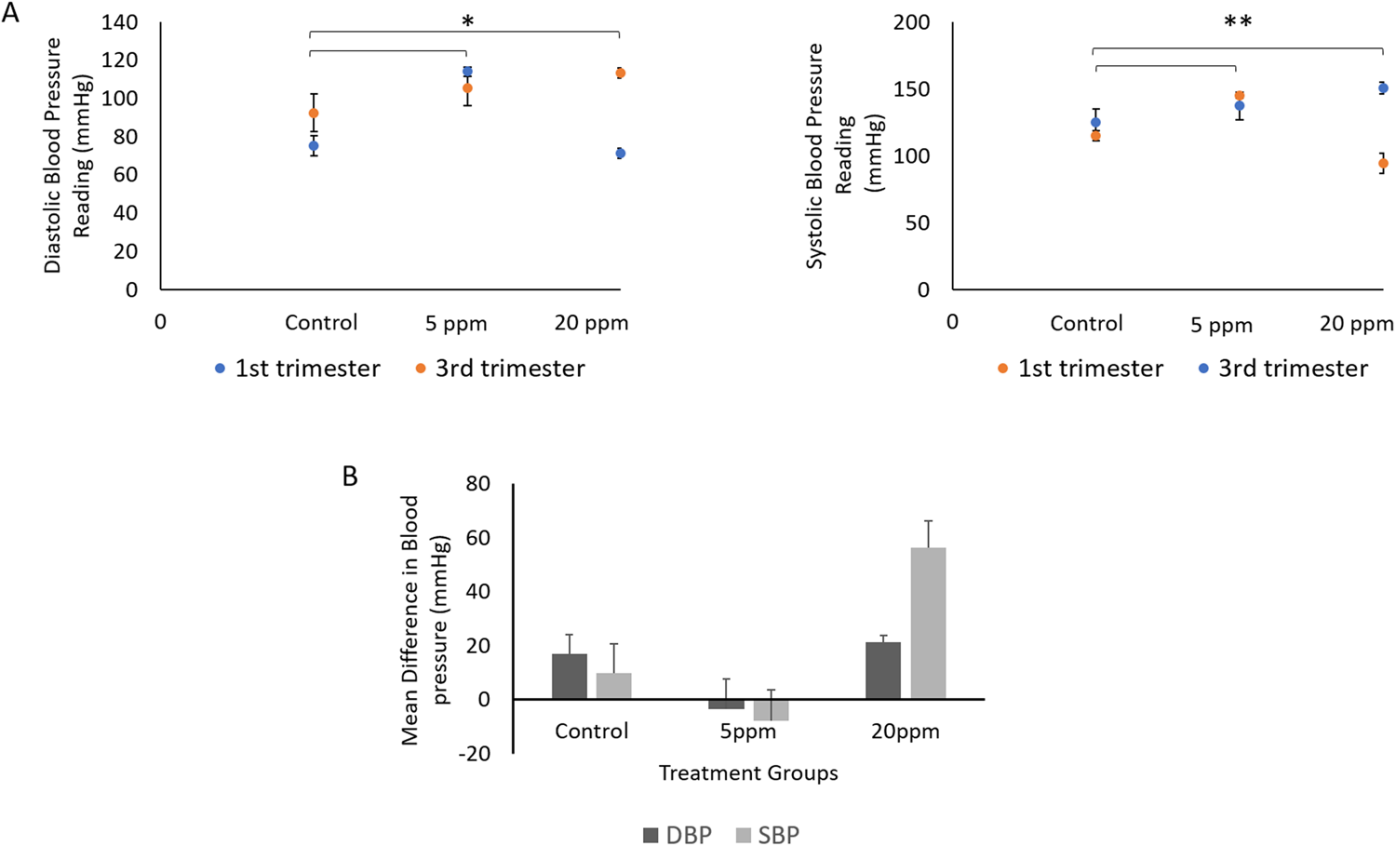
BPA exposure effects mothers blood pressure. (**A**) Diastolic blood pressure (DBP) and systolic blood pressure (SBP) reading in control, 5 ppm BPA-exposed and 20 ppm BPA-exposed pregnant rat. (**B**) Data are expressed as means of differences DBP and SBP readings from pregnancy day 2 with pregnancy day 18 compared to control. Significance differences in SBP was observed in both 5 ppm and 20 ppm BPA exposed pregnant rat with control pregnant rat. Meanwhile no significance differences was observed between all groups. (n = 5). (*p < 0.05; **p < 0.01; ***p < 0.001).

In the pregnant rat, systolic blood pressures (SBP) and diastolic blood pressure (DBP) were measured at PD2 and PD18 (Fig. 3A). The difference in the SBP and DBP were found to be significant within the BPA-exposed groups (p < 0.05). The results indicated that both SBP and DBP significantly increased during pregnancy in both groups of BPA-exposed rats as compared to the control rats (p < 0.05). When the BPA-exposed groups are compared with the control group, the SBP (in the third trimester) was raised up from 125 ± 9.505 mmHg in control rats to 151 ± 4.50 mmHg and 137 ± 10.46 mmHg in the 20 and 5 ppm BPA-exposed rats, respectively (p = 0.009). DBP progressively increased significantly during third trimester in the BPA-exposed groups from 92 ± 9.86 mmHg in the control group to 113.00 ± 2.54 mmHg and 105.00 ± 9.35 mmHg in the 20 and 5 ppm of BPA, respectively (p = 0.028, p = 0.007).

The differences between BP reading from PD2 and PD18 is shown in Fig. 3B. The results indicated that SBP increased significantly in the 20 ppm (56.4 ± 9.902 mmHg) BPA-exposed pregnant rats in comparison to control (10.0 ± 10.775 mmHg) pregnant rats. Meanwhile, DBP also showed the similar pattern in SBP increment of 20 ppm (21.0 ± 2.534 mmHg) BPA-exposed pregnant rats in comparison to control (17.0 ± 6.979 mmHg) pregnant rats.

**Figure 4:**
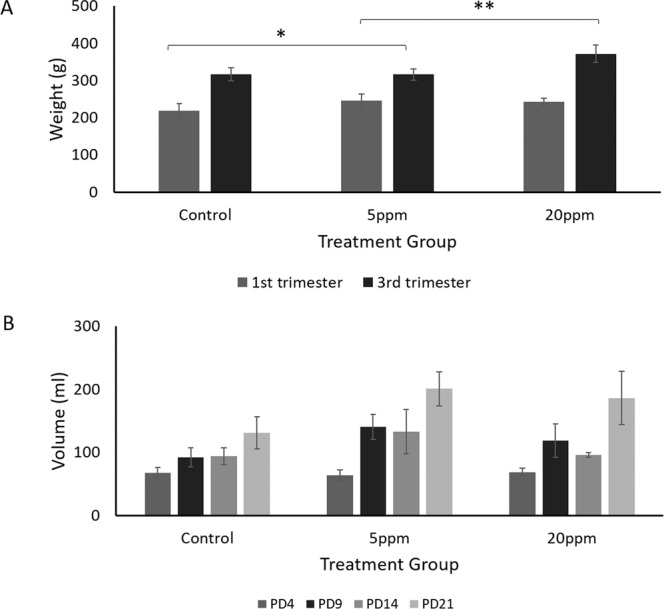
BPA exposure during pregnancy affect weight gained of mothers. (**A**) Body weight of (wt) and (**B**) drinking pattern of control, 5 ppm BPA-exposed and 20 ppm BPA-exposed pregnant rats. Data are expressed as means. (n ≥ 5).*p < 0.05; **p < 0.01.

**Figure 5:**
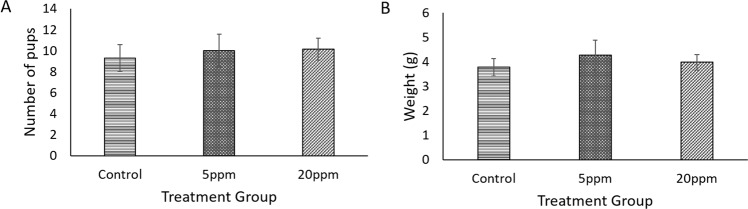
BPA has no effect on number and weight of foetuses. (**A**) Number of Foetuses (**B**) Birth Weight of Foetuses (wt). Data are expressed as means. (n > 5 mothers); more than one offspring were selected per litter.

All mothers significantly gained weight from PD2 to PD18 regardless of whether they were given vehicle control or being exposed to BPA (Fig. 4A). This suggested that all mothers are healthy throughout the treatment. As compared to the control, weight gained of BPA-exposed rats with 5 ppm (54 ± 19.61 g) was found to be significant (p = 0.025) and 20 ppm (135 ± 39.95 g) was nearly significant (p = 0.053). Interestingly, the significant difference in weight gained was observed between both BPA-exposed groups (p = 0.001). Apart from that, no significant differences were observed for the drinking pattern in either control or BPA-exposed rats (Fig. 4B). Although changes in the foetuses weight and size were observed, however, there were no significant differences in both parameters between control and BPA-exposed foetuses (Fig. 5A,B, respectively).

#### Bisphenol A prenatal exposure alter foetuses cardiac miRNAs involved in development and diseases

**Figure 6:**
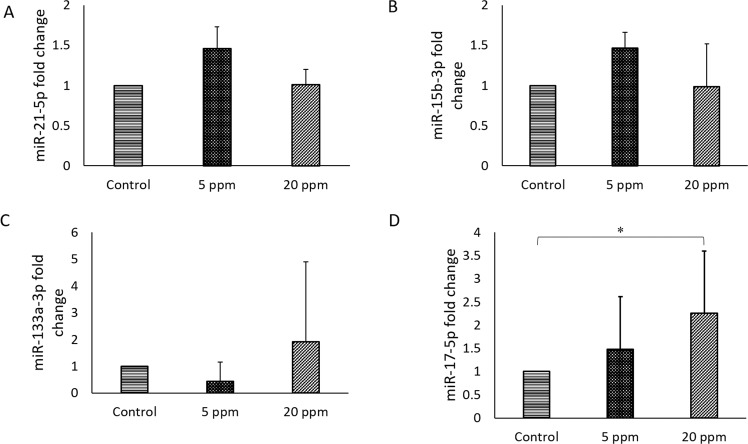
miR-17-5p was upregulated in foetuses heart of BPA-exposed pregnant rats. miRNA expression of (**A**) miR-21-5p (**B**) miR-15b-3p (**C**) miR-133a-3p (**D**) miR-17-5p were measured in the heart of foetuses of control and BPA-exposed pregnant rats. All the miRNA have been reported involved in cardiac development. Data are expressed as means. (n = 5 rats per group). *p < 0.05; **p < 0.01; ***p < 0.001.

miR-17-5p, -21-5p and -133a-3p have been reported to be upregulated during the incidence of cardiac disease. These miRNAs are important in cardiomyocytes proliferation and development^45^. In this study, significant elevation of miR-17-5p expression was observed in 5 ppm (1.50 ± 1.14) and 20 ppm (2.25 ± 1.34) compared to the control (p = 0.027) (Fig. 6).

**Figure 7:**
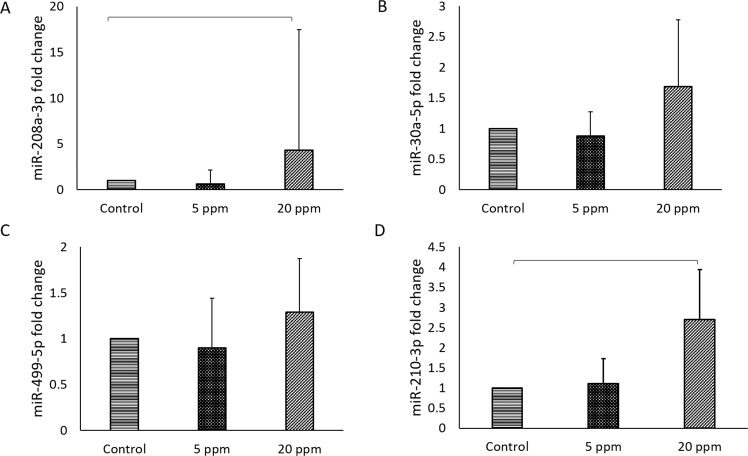
miR-208a-3p and miR-210-3p were upregulated in foetuses heart of BPA-exposed pregnant rat. (**A**) miR-208a-3p (**B**) miR-30a-5p (**C**) miR-499-5p (**D**) miR-210-3p expression were measured in foetuses heart of control and BPA-exposed pregnant rats. Altered expression in these miRNAs have been associated with increase risk of cardiac disease development. Data are expressed as means. (n = 5 rats per group). *p < 0.05; **p < 0.01; ***p < 0.001.

In addition, significant upregulation of miR-208a-3p and -210-3p were seen in BPA-treated group compared to the control group (Fig. 7). miR-208a-3p has been observed to be upregulated during muscle injury and cardiac hypertrophy. miR-208a-3p increased only in 20 ppm BPA (4.30 ± 13.19, p = 0.039). A similar finding was observed in the expression of miR-210-3p with an upregulation in 20 ppm BPA (2.70 ± 1.25, p = 0.039).

**Figure 8:**
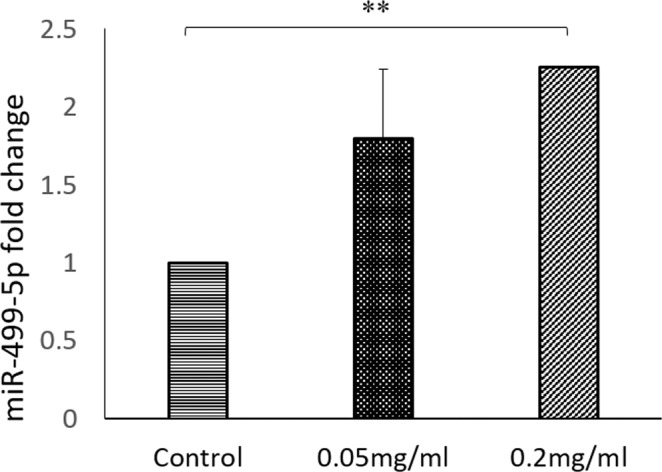
BPA exposure has significant effect on miR-499-5p expression of mother’s heart. miR-499-5p is related to myocardial infarction. Data are expressed as means. (n = 5 rats per group).

In the mother’s heart tissues, only miR-499-5p was significantly elevated in both BPA-exposed groups, 5 ppm (1.79 ± 0.60) and 20 ppm (2.25 ± 0.45) (p = 0.005) (Fig. 8). The significant values of miRNA expression are as tabulated in Table 2.

**Table 2:** Expression miRNAs important in heart development and diseases of foetuses heart from BPA-exposed pregnant rats.

miRNAs	BPA concentration (ppm)	Mother	p-value	Foetus	p-value
miR-15b-3p	5	0.400 ± 0.339	0.491	1.463 ± 0.201	0.058
	20	0.494 ± 0.284		0.982 ± 0.534	
miR-17-5p	5	0.814 ± 0.368	0.268	1.475 ± 1.139	0.027*
	20	0.793 ± 0.369		2.257 ± 1.343	
miR-21-5p	5	0.806 ± 0.278	0.371	1.459 ± 0.270	0.068
	20	0.609 ± 0.222		1.009 ± 0.189	
miR−133a-3p	5	1.489 ± 3.391	1.000	0.431 ± 0.723	0.073
	20	1.817 ± 2.880		1.916 ± 2.974	
miR-208a-3p	5	1.305 ± 43.717	0.985	0.656 ± 1.517	0.039*
	20	1.932 ± 48.973		4.301 ± 13.191	
miR-499-5p	5	1.794 ± 0.599	0.005*	0.900 ± 0.584	0.593
	20	2.255 ± 0.448		1.291 ± 0.241	
miR-210-3p	5	0.840 ± 0.585	0.679	1.104 ± 0.619	0.039*
	20	0.977 ± 0.412		2.698 ± 1.249	
miR-30a-5p	5	1.588 ± 2.677	0.564	0.874 ± 0.397	0.273
	20	2.262 ± 3.411		1.683 ± 1.096	

Values presented as mean ± SEM, n = 5. p-values generated from Non parametric Study Kruskal Wallis Test.

*Indicates treatment significantly different from baseline, p < 0.05.

#### Cardiac fibrosis and expression of cardiac troponin I in BPA-exposed foetal heart

**Figure 9:**
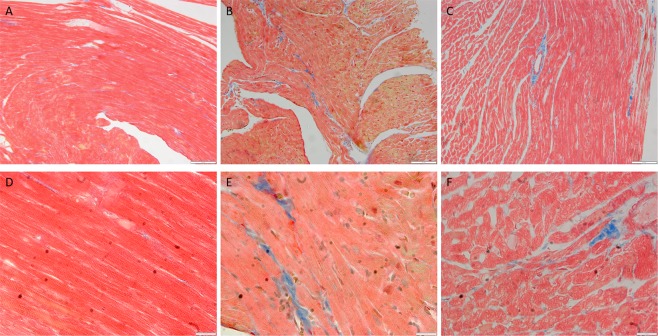
BPA exposure effects mothers hearts Masson Trichrome staining. Representative of heart section of control (**A**) and 5ppm and 20ppm BPA-exposed (**B**,**C**) pregnant rats, stained with Masson trichrome (10X magnification). (**D**–**F** represents section in 40X magnification).

**Figure 10:**
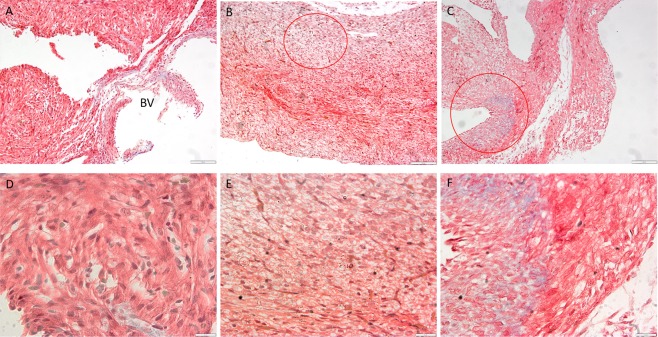
BPA exposure effects foetal hearts Masson Trichrome staining. Representative of heart section of control (**A**) and 5ppm and 20ppm BPA-exposed (**B**,**C**) of foetal heart (10X magnification). (**D–F**) Foetal heart section at PD21 from pregnant rats of control, 5 ppm BPA and 20 ppm BPA-exposed (40X magnification). Note: BV = Blood vessel. The red circle represents sign of fibrosis with bluish stained.

**Figure 11:**
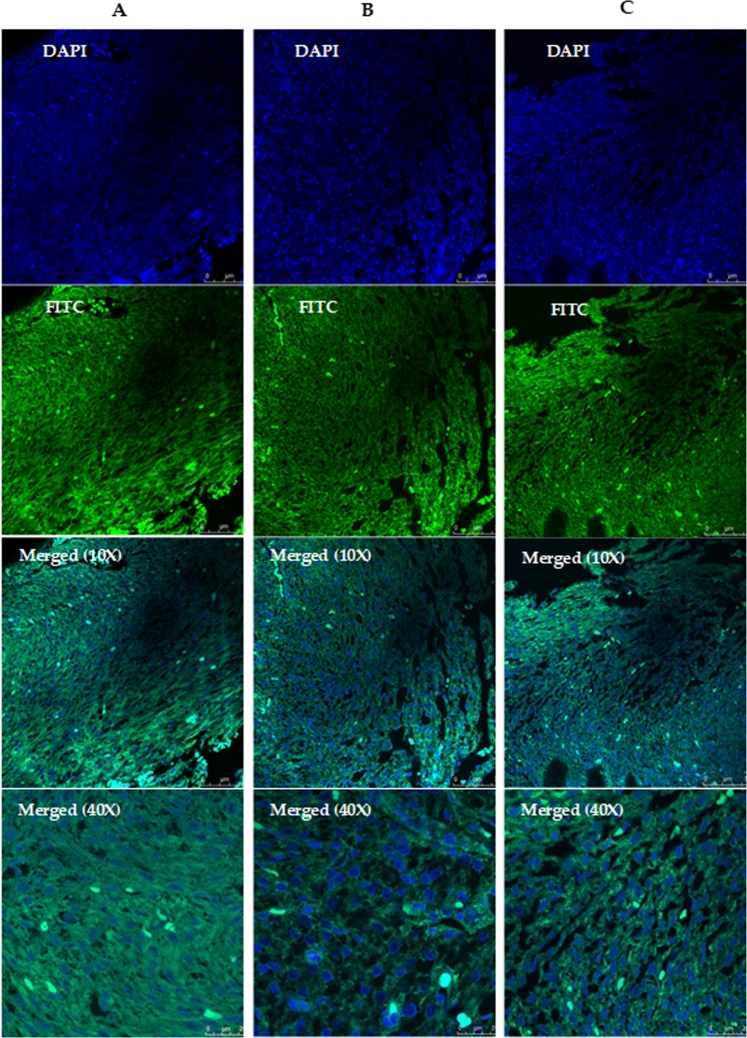
BPA exposure effects foetal cardiac troponin I (cTnI) expression. Immunofluorescence staining of foetal heart section subjected to cardiac troponin I expression. (**A**) control foetal heart, (**B**) 0.05 mg/ml BPA-exposed, (**C**) 0.2 mg/ml BPA-exposed foetal heart, stained with DAPI and FITC. ZF 2.6 (n = 8). Scale bar 25 µm and 75 µm.

In here, non-prominent presence of fibrosis was observed in heart of pregnant rats of BPA-treated groups compared to control (Fig. 9). As for foetus, Masson trichrome staining further confirmed the appearance of fibrosis observed in H&E staining (Fig. 10), BPA-exposed foetal heart showed signs of fibrosis (in red circle) based on the observation. However, more prominent sign of fibrosis was confirmed in 20 ppm of BPA-exposed foetal heart compared to 5 ppm and control. The expression of cardiac troponin I (cTnI) was reduced in BPA-exposed foetal heart compared to the control of foetal heart (Fig. 11).

### Discussion

Many *in vivo* and *in vitro* studies have investigated the detrimental effects of BPA exposure in the early development of different organs and cell types. Recently, BPA has been shown to affect the normal function of *in vitro* fetus heart model^46^. In the liver, abnormalities in its function and damage were reported in rat offspring of BPA-exposed pregnant rats^47^. The study demonstrated that the liver abnormalities and damage were only observed at the postnatal of week 21 offspring from BPA-exposed pregnant rats. Meanwhile, in our study, we showed that prenatal exposure of 5 ppm and 20 ppm BPA had no significant effects on the liver morphology of the foetuses and pregnant rats. The structure of the liver portrays normal hepatic function suggesting that prenatal BPA exposure at these doses does not exert hepatotoxicity. Although the doses used in this study are high (5 ppm and 20 ppm), these high BPA concentrations have been suggested as estimated daily intake based on data extrapolated from urinary BPA levels^48^. A previous study on reproductive organs observed no increment of cancer in 0.015-75 ppm and 750–7500 ppm of BPA exposure^49^. However, other studies documented that BPA foetal exposure at the range of environmental (less than 1 ppm) and high concentration (15 to 150ppm) showed a correlation of BPA concentration with its detrimental effects on the embryos ^50,51^. Similarly, this study demonstrated the effect of BPA in both the mothers and foetus hearts, whereby histology on the heart of the BPA-exposed pregnant rats showed injury in the muscle which could be due to the necrotic process or cardiomegaly. Meanwhile, in the foetuses, histology examination showed a sign of fibrosis compared to the control. These suggested that BPA might give an impact on foetal development within the chosen concentrations.

Nevertheless, this study revealed a correlation between positive weight gain in the BPA-exposed groups with raised SBP readings throughout the treatment (Fig. 3). Increased body weight has been identified as a risk factor for hypertension development during pregnancy^52^. This could be due to the rise in cardiac output and heart rate. Recently, researchers have reported the association of CVD with hypertension in the BPA-exposed individual. These two chronic diseases are very common as one of the public health problems^53^. Thus, Han et. al (2016) conducted a study to investigate the association between BPA exposure and CVD development of in a randomized clinical trial. The study observed an increment in SBP with 4.5 mmHg after the exposure to BPA. Our results were in agreement with those findings where we showed changes in BP in BPA-exposed pregnant rats compared to the control pregnant rats. Although the mechanisms of associations between BPA, hypertension and CVD were unclear, it could occur due to several factors such as endocrine disruptor, oxidative stress and inflammation induction, links with other chronic diseases and epigenetic changes.

As shown in Fig. 4A, all rats gained weight throughout the treatment period. However, a significant weight gain was observed after the exposure of 5 ppm BPA compared to the control pregnant rats (p = 0.025). This observation was in line with few reported studies, which demonstrated an increment of weight in those exposed with BPA^8,54,55^. Exposure to an endocrine-disrupting chemical such as BPA promotes adipogenesis and weight gain. This made BPA as a potential determinant of obesity^56^. This study also confirms the obesogenic impacts on male mice after BPA exposure. In contrast, another study reported no significant differences in weight between vehicle control and BPA groups^57^. The study was conducted on Sprague-Dawley rats from gestational day 6 until postnatal day 90. They observed no significant increment on the gestational length and bodyweight of the pups exposed to BPA. This is in line with the results reported in this study (Fig. 5B). They interpreted that certain BPA dosage might not affect the endpoint results although few previous studies reported on the effect of BPA exposure on weight gain.

Epigenetic modifications are known to be related to diseases such as CVD and there are many studies have already linking epigenetic changes with BPA exposure^58^. Based on these findings, we further investigated the impact of BPA exposure on miRNAs. Three out of eight targeted cardiac-related miRNAs exhibited altered expression in foetuses of BPA-exposed group. This observation is in agreement with a study that was conducted by Barry et. al. (2013) in investigating the toxicity levels of BPA in rats^57^. Previous study observed overexpression of miR-21 in BPA-induced 3T3-L1 cells as it has been reported to be an important gene in the regulation of insulin and glucose^59^. Nevertheless, previous study also reviewed the abnormally expressed miR-21–5p in cancer development such as breast, lungs and colorectal^60^.

miR-133a-3p and -208a-3p are involved in cardiomyocytes proliferation and differentiation^45^. As we were focusing on the possibility of the offspring to develop cardiac disease, miRNAs related to cardiac disease were chosen. Interestingly, miR-208a-3p was elevated in foetuses with 20 ppm BPA-exposed pregnant rat (p = 0.039). miR-208a-3p is involved in cardiac contractility and function, where the expression of this gene in response to modulation of myosin heavy chain results in the upregulation of βMHC. miR-208a-3p is a cardiac-specific miRNA and has been reported to be an important miRNA in cardiomyocyte hypertrophy and fibrosis^3^. This gene is absent in healthy subject thus, the elevation of its expression suggested a modification in the heart of the foetus exposed to BPA.

This study also focuses on the impact of BPA exposure on the heart of mothers and foetuses, by H&E, Masson trichrome (MT) staining, cTnI expression and cardiac miRNA profile. Our findings showed that elevated expression of miR-208-3p (in foetal heart) and miR-499-5p (in mothers heart) after exposure to BPA could be associated with the expression of cardiac troponin I (cTnI) (Fig. 11). This findings are in agreement with previous reported studies which associated the upregulation of miR-208a and miR-499 with cTnI expression^7,61,62^.

In agreement with our findings, miR-17-5p is elevated in foetus-exposed to BPA, thus, suggested the possibility of the offspring to develop heart disease later in life. There was study documented on a role of miR-17 in liver and cardiac fibrosis^63^. miR-210-3p is significantly expressed in the BPA-exposed foetus heart, which suggests that this miRNA has an impact on angiogenesis and fibrosis^64^. In parallel, a study reported similarities between foetal and failing heart in which elevated miR-210-3p was observed, indicating the prevalence of heart failure development^65^. In our findings, this gene is significantly altered in 20 ppm of BPA-exposed foetus heart. Moreover, our H&E and MT staining indicated fibrosis in both BPA exposed groups compared to control group. However, the fibrosis was more prominent in 20 ppm BPA. Previously, BPA shown to upregulate the expression of marker for fibrosis such as interleukin-6 (IL-6) and collagen type 1^66,67^. In here, our result is the first to suggest that BPA exposure during prenatal life may altered cardiac miRNAs and induced fibrosis.

### Conclusions

There is increasing evidence that exposure to BPA during the intrauterine environment may play a significant role in the global health problems specifically in the development of CVD. BPA has the potential to induce obesity and hypertension in the exposed individual during pregnancy. As the foetus is partially protected from the adverse effect of environmental conditions, in utero exposures of BPA may give impact to the foetus indirectly. Furthermore, our data suggest that altered miRNA expression in the foetus do affect the heart tissues. Thus further study need to be done on understanding whether these changes seen in BPA-exposed foetus could increase the risk of CVD in offspring later in life.

## Limitation

There are some limitations to this study. Firstly, this study focused on the impact of miRNAs expression in the foetal hearts only and does not extend until in postnatal life. However, the expression during postnatal life could be influenced by other factors such as environmental and nutrient intake that could interfere with the overall findings. Secondly, the number of the sample size used in this study is limited to 5. The expression could be more significant if the sample size is bigger than 5.

### Ethics approval

This study was conducted in accordance of relevance guidelines and regulations, and approved by Universiti Teknologi MARA (UiTM) Committee of Animal Research and Ethics (Approval Number UiTM CARE: 222/7/2017 (8/12/2017)).

## Supplementary information

Supplementary Information.

## Data Availability

The datasets used and/or analysed in this study are available from the corresponding author on reasonable request.
