# Supplementary material for: The impacts of intrauterine Bisphenol A exposure on pregnancy and expression of miRNAs related to heart development and diseases in animal model
Source: Sci Rep. 2020 Apr 3;10:5882. doi: 10.1038/s41598-020-62420-1 (PMC7125099; doi:10.1038/s41598-020-62420-1)
Supplement: Supplementary file 1 — Supplementary Information. [file 41598_2020_62420_MOESM1_ESM.docx]

Supplementary Data

**The impacts of intrauterine Bisphenol A exposure on pregnancy and expression of miRNAs related to heart development and diseases in animal model**

**Zatilfarihiah Rasdi^1,2^, Roziana Kamaludin^3^, Sharaniza Ab. Rahim^4^, Mohd Hafiz Dzarfan Othman^3^, , Syed Baharom Syed Ahmad Fuad^4^, Rosfaiizah Siran^4^, Noor Shafina Mohd Nor^4,5^**, **Narimah Abdul Hamid Hasani^4^, Siti Hamimah Sheikh Abdul Kadir^1,4,5*^**

^1^Institute of Medical Molecular Biotechnology, Faculty of Medicine, Universiti Teknologi MARA, Cawangan Selangor, 47000 Sungai Buloh, Selangor, Malaysia.

^2^Centre of Preclinical Sciences Studies, Faculty of Dentistry, Universiti Teknologi MARA, Cawangan Selangor, 47000 Sungai Buloh, Selangor, Malaysia

^3^Advanced Membrane Technology Research Centre (AMTEC), Universiti Teknologi Malaysia, 81310 Skudai, Johor, Malaysia

^4^Faculty of Medicine, Universiti Teknologi MARA, Cawangan Selangor, 47000 Sungai Buloh, Selangor, Malaysia

^5^Institute for Pathology, Laboratory and Forensic Medicine (I-PPerForM), Universiti Teknologi MARA, Cawangan Selangor, 47000, Sungai Buloh, Selangor, Malaysia

*****Correspondence: sitih587@uitm.edu.my; Tel.: +603-6126-5003

Based on the observation, low dose of BPA exposure (1 ppm of BPA) do not show any significant differences in BP of pregnant rats (Figure 1) and also histology of muscle in foetal heart exposed to BPA (Figure 2).


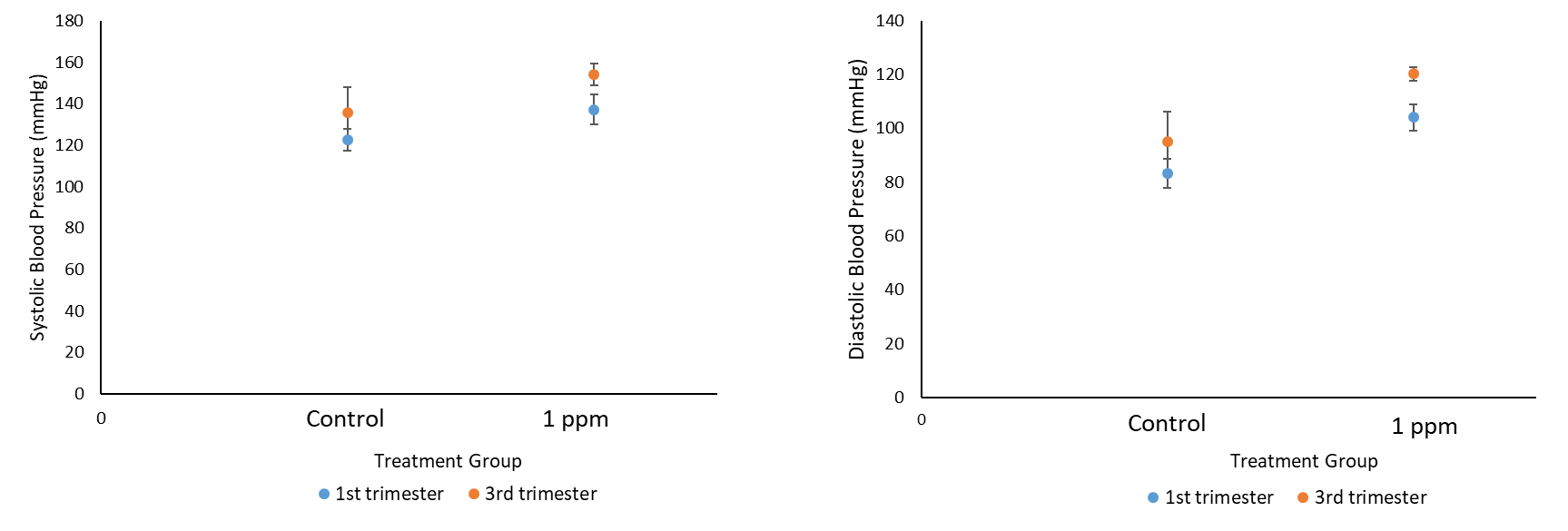


**Figure 1.** **BPA exposure does not effect mothers blood pressure**. Diastolic blood pressure (DBP) and systolic blood pressure (SBP) reading in control and 1 ppm BPA-exposed pregnant rat. No significance differences in SBP and DBP was observed in 1 ppm BPA exposed pregnant rat with control pregnant rat. (n=4).


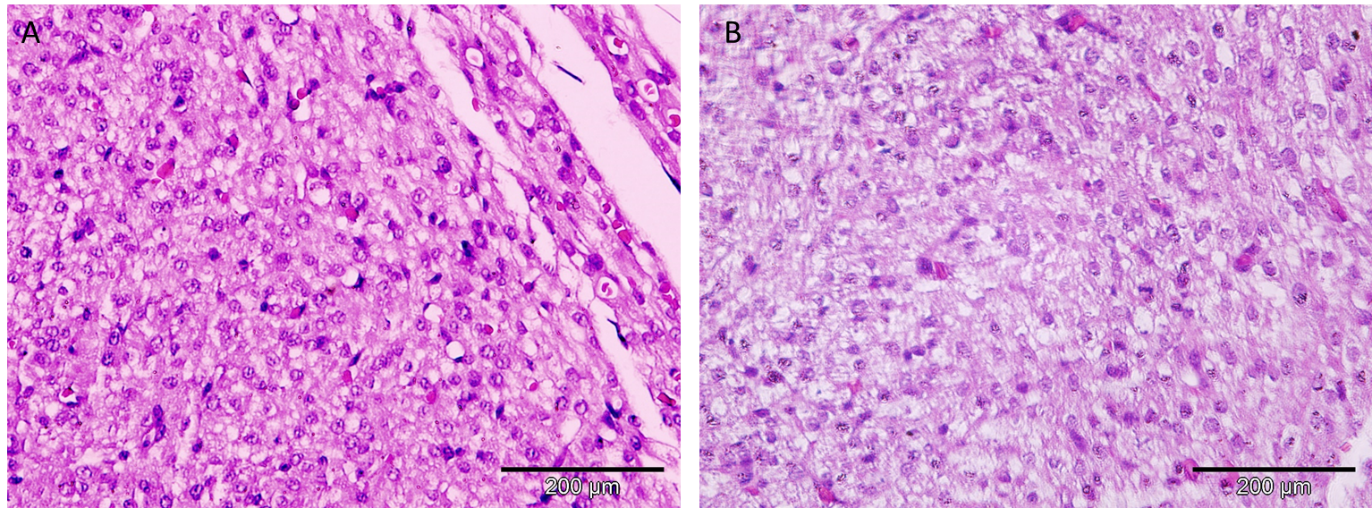


**Figure 2 BPA exposure does not effect foetal hearts histology**. Representative of heart section of control (A) and 1ppm BPA-exposed (B) foetal heart stained with haematoxylin and eosin dye (40X magnification).
